# Deep learning-based segmentation of the thorax in mouse micro-CT scans

**DOI:** 10.1038/s41598-022-05868-7

**Published:** 2022-02-02

**Authors:** Justin Malimban, Danny Lathouwers, Haibin Qian, Frank Verhaegen, Julia Wiedemann, Sytze Brandenburg, Marius Staring

**Affiliations:** 1 Department of Radiation Oncology, University Medical Center Groningen, University of Groningen, 9700 RB Groningen, The Netherlands; 2 Department of Radiation Science and Technology, Faculty of Applied Sciences, Delft University of Technology, 2629 JB Delft, The Netherlands; 3 Department of Medical Biology, Amsterdam University Medical Centers (Location AMC) and Cancer Center Amsterdam, 1105 AZ Amsterdam, The Netherlands; 4 Department of Radiation Oncology (MAASTRO), GROW School for Oncology and Developmental Biology, Maastricht University Medical Center, 6229 ER Maastricht, The Netherlands; 5 Department of Biomedical Sciences of Cells and Systems-Section Molecular Cell Biology, University Medical Center Groningen, University of Groningen, 9700 RB Groningen, The Netherlands; 6 Department of Radiology, Leiden University Medical Center, 2333 ZA Leiden, The Netherlands

**Keywords:** Preclinical research, Computed tomography, Physics

## Abstract

For image-guided small animal irradiations, the whole workflow of imaging, organ contouring, irradiation planning, and delivery is typically performed in a single session requiring continuous administration of anaesthetic agents. Automating contouring leads to a faster workflow, which limits exposure to anaesthesia and thereby, reducing its impact on experimental results and on animal wellbeing. Here, we trained the 2D and 3D U-Net architectures of no-new-Net (nnU-Net) for autocontouring of the thorax in mouse micro-CT images. We trained the models only on native CTs and evaluated their performance using an independent testing dataset (i.e., native CTs not included in the training and validation). Unlike previous studies, we also tested the model performance on an external dataset (i.e., contrast-enhanced CTs) to see how well they predict on CTs completely different from what they were trained on. We also assessed the interobserver variability using the generalized conformity index ($\hbox {CI}_{\mathrm{gen}}$) among three observers, providing a stronger human baseline for evaluating automated contours than previous studies. Lastly, we showed the benefit on the contouring time compared to manual contouring. The results show that 3D models of nnU-Net achieve superior segmentation accuracy and are more robust to unseen data than 2D models. For all target organs, the mean surface distance (MSD) and the Hausdorff distance (95p HD) of the best performing model for this task (nnU-Net 3d_fullres) are within 0.16 mm and 0.60 mm, respectively. These values are below the minimum required contouring accuracy of 1 mm for small animal irradiations, and improve significantly upon state-of-the-art 2D U-Net-based AIMOS method. Moreover, the conformity indices of the 3d_fullres model also compare favourably to the interobserver variability for all target organs, whereas the 2D models perform poorly in this regard. Importantly, the 3d_fullres model offers 98% reduction in contouring time.

## Introduction

Preclinical in vivo studies using small animal models serve as an essential experimental system to evaluate potential benefits and radiobiological implications of treatment strategies before clinical implementation. They play an integral role in modelling the disease, disease treatment, and response to treatment under clinically relevant radiation exposure conditions that can potentially translate to improvements in therapeutic outcomes. Over the years, extensive research has been done to develop small animal imaging and irradiation platforms for X-ray therapy^1–5^. Commercial irradiation units such as the Small Animal Radiation Research Platform (SARRP, Xstrahl Ltd., Camberley, UK) and X-RAD SmART+ (PXI North Branford, CT, USA) are also available, providing image-guided irradiations representative of clinical scenarios^1,6^. Recently, research groups have also started to adopt these technologies for proton preclinical research by integrating them with a proton beamline to perform image-guided proton irradiations^7–9^.

The preclinical irradiation workflow involves the following stages: (1) animal set-up, (2) image acquisition, (3) organ contouring, (4) irradiation planning, and (5) radiation delivery. The process begins with administration of anaesthesia to immobilize the animal and is followed by placement in the irradiation position. Then, 3D volumetric scans of the animal are acquired using micro computed tomography (micro-CT) or other imaging modalities. These images are used to identify the shape and location of target volumes and delineate their boundaries. Then, an irradiation plan is created, and dose distributions are calculated. Once the irradiation objectives and dose constraints are met, the plan is delivered to the animal. For image-guided small animal irradiation, this entire process is preferably carried out consecutively in a single treatment session, which typically lasts for 20–90 min, during which the animal is continuously maintained under anaesthesia^10^. However, prolonged exposure of rodents to anaesthetic agents has been shown to influence physiological parameters which can potentially affect the outcome of experiments^11,12^. Therefore, a fast irradiation workflow is warranted.

One of the most time-consuming tasks in preclinical image-guided irradiation workflow is organ contouring. Traditionally, the organ contours are created manually by a biologist. This approach is not ideal and can be very tedious since in a single study a large group of animals may be irradiated. As an example, in studies of normal tissue damage well over 100 animals were irradiated in a single session^13^. In this study, organ contours of only five animals were made upon which irradiation plans for the entire population were based. Automating organ contouring not only reduces the overall workload for preclinical irradiations, but also allows plans to be created tailored to individual animals. This can lead to a better predictive value of preclinical studies and in effect, the number of animals required to meet the objectives of the study may also be reduced. Moreover, individualized contours are essential for animals implanted with orthotopic tumors, which exhibit greater morphological variation compared to the normal tissues.

Over the years, several methods have been developed to automate and speed up the contouring process. One of the most widely used autocontouring techniques for biomedical applications is the atlas method^14^. Several whole-body atlases of mouse anatomy have been constructed such as the MOBY phantom^15^ and the Digimouse atlas^16^ which were based on a single reference animal. However, some studies have pointed out that this approach (“classic single atlas’) produces inferior segmentation accuracy as it exhibits strong bias towards the selected atlas, and it cannot capture realistic body deformations caused by posture, weight, fat amount, and body length variations^17^. To address this problem, deformable atlases, which can adapt arbitrary poses and adjust organ anatomy based on changes in body weight, length, and fat amount, have been proposed^18,19^. Another potential solution to compensate for individual variations is the use of multiple atlases constructed from different subjects. The multi-atlas-based image segmentation (MABIS) algorithm developed by van der Heyden et al. was able to complete the contouring process in a relatively short time ($\sim$ 12 mins) and generated accurate segmentations for organs with sharp boundaries, but manual corrections were needed for less sharp ones^20^.

Although atlas-based segmentation methods are generally faster than manual contouring, the effective runtime of the segmentation task is still considerable, and it may be further reduced using deep learning techniques. In particular, convolutional neural networks (CNN) have shown encouraging results in human organ segmentation. Several studies have demonstrated that deep learning-based segmentation yielded more consistent and more accurate results than atlas-based methods for clinical images^21,22^. It also outperformed the atlas-based methods in terms of speed^23^. CNNs have also found applications in preclinical image segmentation. Van der Heyden et al. used a two-step 3D U-Net model to automatically delineate the skeletal muscle in the lower limb of mice which was shown to be 150 times faster than manual segmentation^24^. For multi-organ segmentation, Wang et al. developed a 3D two-stage deeply supervised network (TS-DSN) for delineation of major organs in the torso of a mouse with an inference time of less than 2 s^25^. More recently, Schoppe et al. developed a deep learning pipeline based on a 2D U-Net-like network called AIMOS (AI-based Mouse Organ Segmentation), which achieved an inference time of 830 ms^26^. Both models showed superior segmentation accuracy compared to existing studies on atlas-based methods. Moreover, AIMOS outperformed TS-DSN except for heart segmentation.

In this work, we trained and validated the 2D and 3D U-Net architectures of no-new-Net (nnU-Net) for segmentation of organs in the mouse thorax and compared their performance to the state-of-the-art AIMOS method. We used only native CT scans for the training and validation phase, and we evaluated the trained models’ accuracy using an independent testing dataset (i.e., native CTs not included in the training and validation). Unlike previous works, we also tested the trained models against an external dataset (i.e., contrast-enhanced CTs), which does not share the same properties such as the mouse strain and image acquisition parameters as the training data. The external dataset was used to investigate the robustness of the neural networks to datasets that are completely different from what they were trained on. Moreover, we thoroughly compared the accuracy of the automated contours relative to human performance by evaluating the generalized conformity index among three observers. Lastly, we assessed by how much these neural networks can shorten the contouring time compared to manual contouring in order to improve the efficiency of the irradiation workflow. For this segmentation task, we used a publicly available mouse micro-CT dataset^27^, and we provide new annotations by two observers for the entire native CT and a subset of the contrast-enhanced CT datasets. These include spinal cord and separate left and right lung segmentations not provided in the original annotations. We make these annotations publicly available at https://doi.org/10.5281/zenodo.5121272.

## Methods

### Dataset

The micro-CT images used in this work were taken from a public database which includes native and contrast-enhanced 3D whole body scans of mice^27^. Supplementary Table S1 provides a summary of their properties. The native CT dataset is comprised of 140 images from 20 female BALB/c nu/nu mice, with each animal imaged at seven time points spread over a 72-h period. The entire native CT dataset was utilized wherein 105 images were allotted to train and validate the models, while the remaining 35 were used as the test set. To create completely independent training and testing datasets, the native CT scans were divided at the animal level: CT images of 15 animals (105 scans) were used for training and validation, and 5 animals (35 scans) were used for testing. In addition, thirty-five scans from the contrast-enhanced CT (CECT) dataset were taken to serve as a second independent test set to further evaluate the trained model’s generalizability and robustness. This dataset includes CTs of 10 female BALB/cAnNRj-Foxn1nu mice which were also imaged at various time points over a 240-h period. However, only eight animals were considered as two of them did not appear to have contrast enhancement.

In this study, we focused on organs in the thoracic region: heart, spinal cord, right lung and left lung. Both test sets were annotated by three observers. The first observer and two second observers were all trained to follow the same labelling protocol and were supervised by a biologist with more than 5 years of animal contouring experience. All of them used the contouring module of the small animal radiotherapy treatment planning system, SmART-ATP (version 2.0, SmART Scientific Solutions BV, Maastricht, the Netherlands). Delineations by a third observer were taken from the annotations provided together with the CT images^27^. These were resampled to the same voxel resolution of $0.14 \times 0.14 \times 0.14\ \hbox {mm}^3$ using nearest neighbor interpolation.

### Deep learning models

The U-Net is one of the most popular architectures for image segmentation. It is a fully convolutional network (FCN) that has a U-shape, with symmetric encoder (contraction) and decoder (expansion) paths. The encoder performs a series of convolution and pooling operations to extract feature representations from the image that the decoder aims to project onto the pixel space through up-sampling in order to restore the original image size. The U-Net was initially proposed for 2D biomedical image segmentation and has been shown to work well even with small training datasets^28^. This is advantageous for preclinical studies where there are restrictions on the number of animals that can be imaged to build the training data. In this work, we investigated the no-new-Net (nnU-Net) deep learning pipeline^29^, which offers 2D and 3D U-Net-like architectures, and compared its performance to the 2D U-Net-based AIMOS method^26^.

#### no-new-Net (nnU-Net)

The no-new-Net is an out-of-the-box tool for automated image segmentation and has been widely used for clinical data. It is a self-adapting algorithm that follows certain heuristic rules to decide on the training configuration such as the selection of the batch size, patch size, and network topology depending on the dataset provided by the user^29^. It is a fully automated deep learning pipeline, which offers both 2D and 3D U-Net architectures that closely follow the original U-Net design.

In this work, we trained all the available models in nnU-Net from scratch: 2D U-Net (2d), 3D full resolution U-Net (3d_fullres), 3D low resolution U-Net (3d_lowres) and 3D cascade U-Net (3d_cascade). The network architectures for the 2D and 3D models generated by nnU-Net for this dataset are illustrated in Supplementary Fig. S1. nnU-Net generates a 2D U-Net model with a network depth (i.e., number of encoder-decoder levels) of six. It is configured to accept a patch size of $320\times 224$ as input and starts with 32 initial feature channels at the highest layer. The input is downsampled six times in the x and five times in the y direction, resulting in an image size of $5\times 7$ at the bottleneck with 480 feature channels. The 2D U-Net model only operates on coronal slices and implements a batch size of 44 during training. nnU-Net also offers three different 3D models with a network depth of five. The 3D full resolution U-Net model runs on the full resolution data and has been shown to be the best performing configuration among all the nnU-Net models in the segmentation challenges where they have participated^29^. It also starts with the same number of initial feature channels but with a patch size of $128\times 96\times 192$ and a batch size of 2. Downsampling is performed five times in x and z and four times in y, which reduces the feature maps at the bottleneck to $4\times 6\times 6$ with feature channels capped to 320. The 3D low resolution U-Net and 3D cascade U-Net also follow this configuration. However, the 3D cascade U-Net is trained in two stages. The first stage involves training a 3D U-Net on downsampled versions of the training images (3d_lowres). The 3d_lowres model was trained on patches of the dataset at a resolution of $0.19\times 0.19\times 0.19\ \hbox {mm}^3$, and the resulting segmentations are then upsampled to the original voxel spacing of $0.14\times 0.14 \times 0.14\ \hbox {mm}^3$. These segmentations served as the input for the second stage, and training is performed at full resolution. All five folds of the 3d_lowres model must be completed before the second stage of 3D cascade U-Net can be initiated.

#### AI-based mouse organ segmentation (AIMOS)

Recently, Schoppe et al. developed a fully-automated deep learning pipeline dedicated for organ contouring of mice micro-CT images called AIMOS^26^. It is currently the overall best performing algorithm for mouse segmentation. AIMOS provides pre-processing, network training, and post-processing modules, requiring very little intervention from the user. It offers several 2D U-Net-like architectures that only differ in the number of encoder-decoder stages. For this study, the default architecture, UNet-768, was chosen, which employs six encoder-decoder stages with initial 32 feature channels at the highest layer and 768 feature channels at the bottleneck. The network was trained using all slices with a batch size of 32.

### Network training and inference

All neural networks were trained only on native CTs delineated by observer 1. Five-fold cross-validation was performed wherein at each fold, three animals were randomly selected and set aside for validation, while the rest was used for training. The same split configuration was used for all networks. The final predictions were determined through an ensemble voting by taking the average of the predicted probabilities from the five models resulting from training on the individual folds. All experiments for nnU-Net were carried out using an NVIDIA V100 with 12 GB of GPU memory while AIMOS was trained using an NVIDIA Quadro RTX 6000 with 24 GB of GPU memory. The training of the nnU-Net 3d_fullres model with 1000 epochs took approximately 2 days on our computing system. The inference time for both codes was evaluated on the same system (NVIDIA Quadro RTX 6000) to facilitate comparison. For this, we chose to report the average time the models take to preprocess an image, to make an inference and the total runtime. The runtime was measured starting from data preparation up to exportation of the automated contours.

### Evaluation metrics and statistical analysis

The quality of the segmentations generated by each model was evaluated in terms of the Dice similarity coefficient (DSC), mean surface distance (MSD) and 95th percentile Hausdorff distance (95p HD)^30,31^. The DSC measures the degree of overlap between the reference and predicted contours; it increases with overlap and a value of 1 indicates a perfect overlap. MSD and 95p HD give the average and maximum distance measured between closest points on the surface of the contours, respectively. Therefore, smaller values for MSD and 95p HD indicate better correspondence to the ground truth.

To determine whether the difference in the DSC, MSD and 95p HD between the models is significant, a statistical analysis was conducted using a two-tailed Wilcoxon signed rank test with a significance level of $\alpha = 0.05$. The nnU-Net 3d_fullres model was chosen as the base model for comparison because it has been shown to be one of the best performing models in many medical image segmentation tasks^29^. A *p*-value $< 0.05$ is considered statistically significant.

### Interobserver variability (IOV)

The degree of agreement between observer’s delineations was estimated using the generalized conformity index ($\hbox {CI}_{\mathrm{gen}}$). It is defined as the ratio of the sum of the intersecting volumes between all pairs of observers and the sum of union of volumes between the same pairs^32^. The $\hbox {CI}_{\mathrm{gen}}$ is the general form of the Jaccard coefficient^30^ applicable for comparison of more than two delineated volumes. This reduces to the Jaccard coefficient for the two-observer case. Higher values of $\hbox {CI}_{\mathrm{gen}}$ indicate greater similarity between the volumes.

Since the annotations from observer 3 do not include the spinal cord, delineations from only two observers were considered for this organ. There is also no separation of the left and right lungs for observer 3 so the lungs were combined to form the total lung volume for the other two observers to facilitate comparison. The IOV was then compared to the performance of the models against a consensus segmentation among observers for which we will refer to as the reference contour for the rest of the paper. For the spinal cord, only pixels delineated by both observers were included in the consensus, whereas pixels delineated by 2 out of 3 observers were considered for the heart and lungs.

## Results

### Native CT (test set 1)

**Figure 1:**
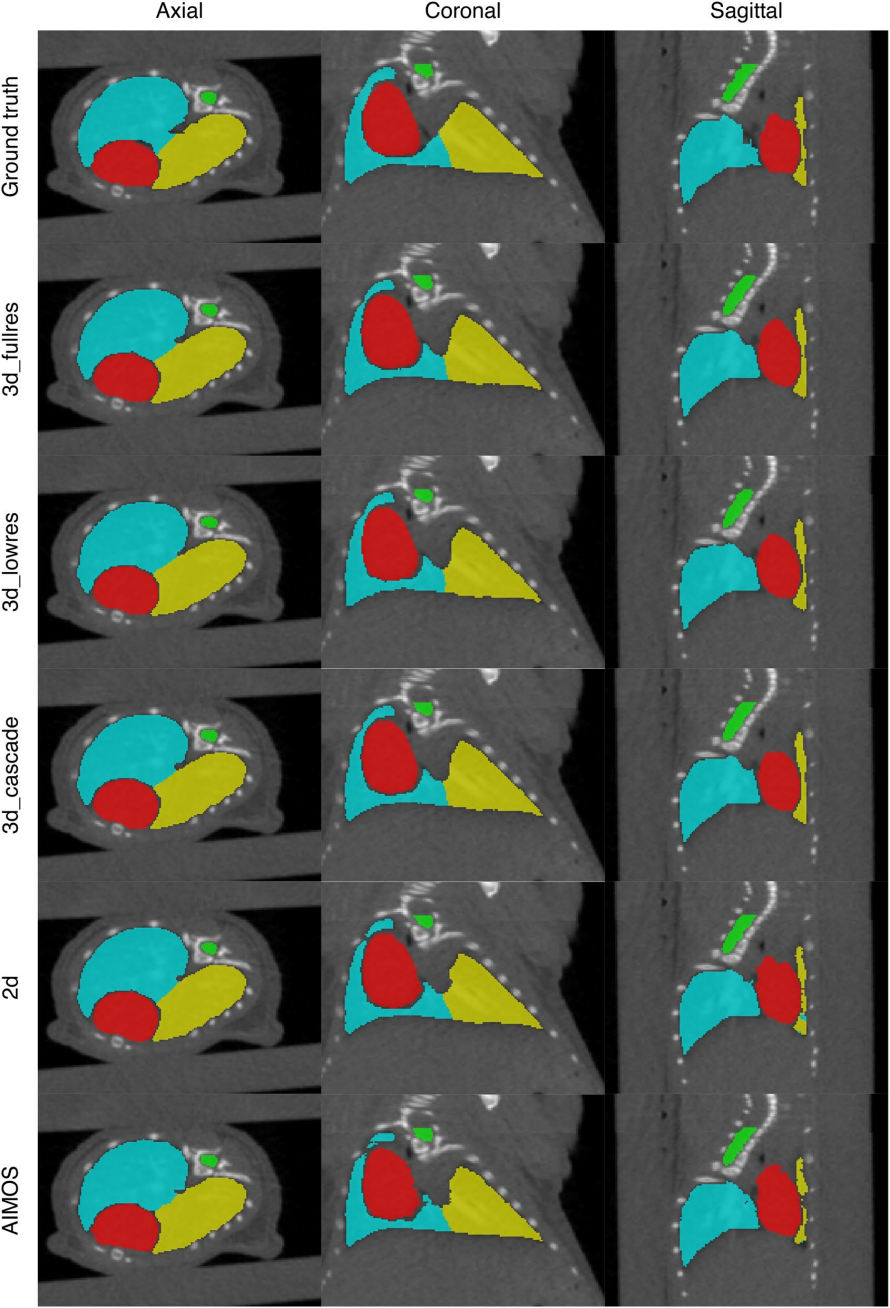
An example segmentation in the axial, coronal and sagittal views for test set 1. The first row shows the manual contours of observer 1 while the succeeding rows are the automated contours generated by each model. Contours in red, green, blue and yellow correspond to the heart, spinal cord, right lung and left lung, respectively.

Figure 1 shows the comparison between the automated and manual contours of observer 1 (i.e., observer who annotated the training data) for an example from test set 1. In general, all neural networks showed correct segmentations for the target organs. The boundaries of the predicted contours appear somewhat smoother than the ground truth. Both the AIMOS and nnU-Net 2d models showed cases wherein parts of the left lung were mislabelled as the right lung or vice versa. The mislabelled pixels can be easily corrected manually as they usually occur in clusters and do not exceed 2% of the total organ volume. Moreover, the nnU-Net 2d model misclassified pixels far from the thorax, which are mostly air cavities labelled either as right lung or left lung. For most cases, only a few pixels were mislabelled by this model, but 2 out of 35 samples exhibited numerous incorrectly labelled pixels.

The boxplots of the DSC, MSD and 95p HD for the heart, spinal cord, right lung, and left lung when compared against the contours of observer 1 are shown in Supplementary Fig. S2, and the mean, median and standard deviation of each metric are given in Table 1. The right and left lungs recorded the highest mean DSC at $0.97\pm 0.01$ followed by the heart and spinal cord at $0.95\pm 0.01$ and $0.91\pm 0.02$, respectively. Using a two-tailed Wilcoxon signed rank test with nnU-Net 3d_fullres as the baseline model for comparison, it was found that the DSC, MSD and 95p HD of nnU-Net 3d_cascade were not significantly different from nnU-Net 3d_fullres for all organs considered. Meanwhile, significant differences ($p<0.05$) on the DSC and MSD values were observed for the other models. They showed slightly inferior performance on these metrics compared to the nnU-Net 3d_fullres model. Nevertheless, all models achieved a mean MSD less than the in-plane voxel size of 0.14 mm while the mean 95p HD were all below 0.60 mm for all organs except the right lung segmentation of nnU-Net 2d. Larger surface distances were observed for this model, resulting from false classifications far from the thoracic region (anaesthesia nozzle). Performing connected component analysis reduced the mean MSD and 95p HD to values similar to what was observed for other structures.

**Table 1:** Comparison of the the segmentation accuracy between different models in terms of the DSC, MSD and 95p HD (mean ± SD and median) for test set 1 annotated by observer 1.

Organ	Algorithm	Model	DSC		MSD (mm)		95p HD (mm)	
			Mean ± SD	Median	Mean ± SD	Median	Mean ± SD	Median
Heart	nnU-Net	3d_fullres	0.95 ± 0.01	0.950	0.08 ± 0.02	0.088	0.30 ± 0.07	0.28
		3d_lowres	0.95 ± 0.01	0.949*	0.09 ± 0.02	0.090*	0.30 ± 0.07	0.28
		3d_cascade	0.95 ± 0.01	0.950	0.09 ± 0.02	0.089	0.31 ± 0.07	0.28
		2d	0.94 ± 0.01	0.945*	0.10 ± 0.01	0.096*	0.33 ± 0.06	0.31*
	AIMOS	UNet-768	0.94 ± 0.01	0.945*	0.10 ± 0.02	0.094*	0.33 ± 0.07	0.31*
Spinal Cord	nnU-Net	3d_fullres	0.91 ± 0.02	0.913	0.04 ± 0.01	0.034	0.28 ± 0.02	0.28
		3d_lowres	0.90 ± 0.02	0.898*	0.04 ± 0.01	0.041*	0.29 ± 0.03	0.28
		3d_cascade	0.91 ± 0.01	0.912	0.03 ± 0.01	0.034	0.28 ± 0.02	0.28
		2d	0.91 ± 0.02	0.907*	0.04 ± 0.01	0.036	0.28 ± 0.02	0.28
	AIMOS	UNet-768	0.90 ± 0.02	0.909*	0.04 ± 0.01	0.036*	0.28 ± 0.02	0.28
Right Lung	nnU-Net	3d_fullres	0.97 ± 0.01	0.970	0.04 ± 0.01	0.036	0.42 ± 0.00	0.42
		3d_lowres	0.97 ± 0.01	0.967*	0.04 ± 0.01	0.040*	0.42 ± 0.00	0.42
		3d_cascade	0.97 ± 0.01	0.970	0.04 ± 0.01	0.035	0.42 ± 0.00	0.42
		2d	0.97 ± 0.01 (0.97 ± 0.01)	0.966*	0.60 ± 0.84 (0.04 ± 0.01)	0.112*	5.29 ± 19.8 (0.42 ± 0.00)	0.42
	AIMOS	UNet-768	0.96 ± 0.01	0.963*	0.05 ± 0.01	0.044*	0.42 ± 0.00	0.42
Left Lung	nnU-Net	3d_fullres	0.97 ± 0.01	0.966	0.04 ± 0.01	0.035	0.53 ± 0.13	0.56
		3d_lowres	0.96 ± 0.01	0.962*	0.04 ± 0.01	0.042*	0.56 ± 0.00	0.56
		3d_cascade	0.97 ± 0.01	0.966	0.04 ± 0.01	0.035	0.53 ± 0.13	0.56
		2d	0.96 ± 0.01	0.965*	0.04 ± 0.02	0.037*	0.54 ± 0.09	0.56
	AIMOS	UNet-768	0.95 ± 0.01	0.956*	0.05 ± 0.01	0.045*	0.56 ± 0.00	0.56

The asterisk (*) indicates a significant difference with the nnU-Net 3d full resolution model according to the Wilcoxon signed rank test with a significance level of $\alpha =0.05$. Values in parentheses correspond to re-calculated metrics after connected component analysis.

### Contrast-enhanced CT (test set 2)

**Figure 2:**
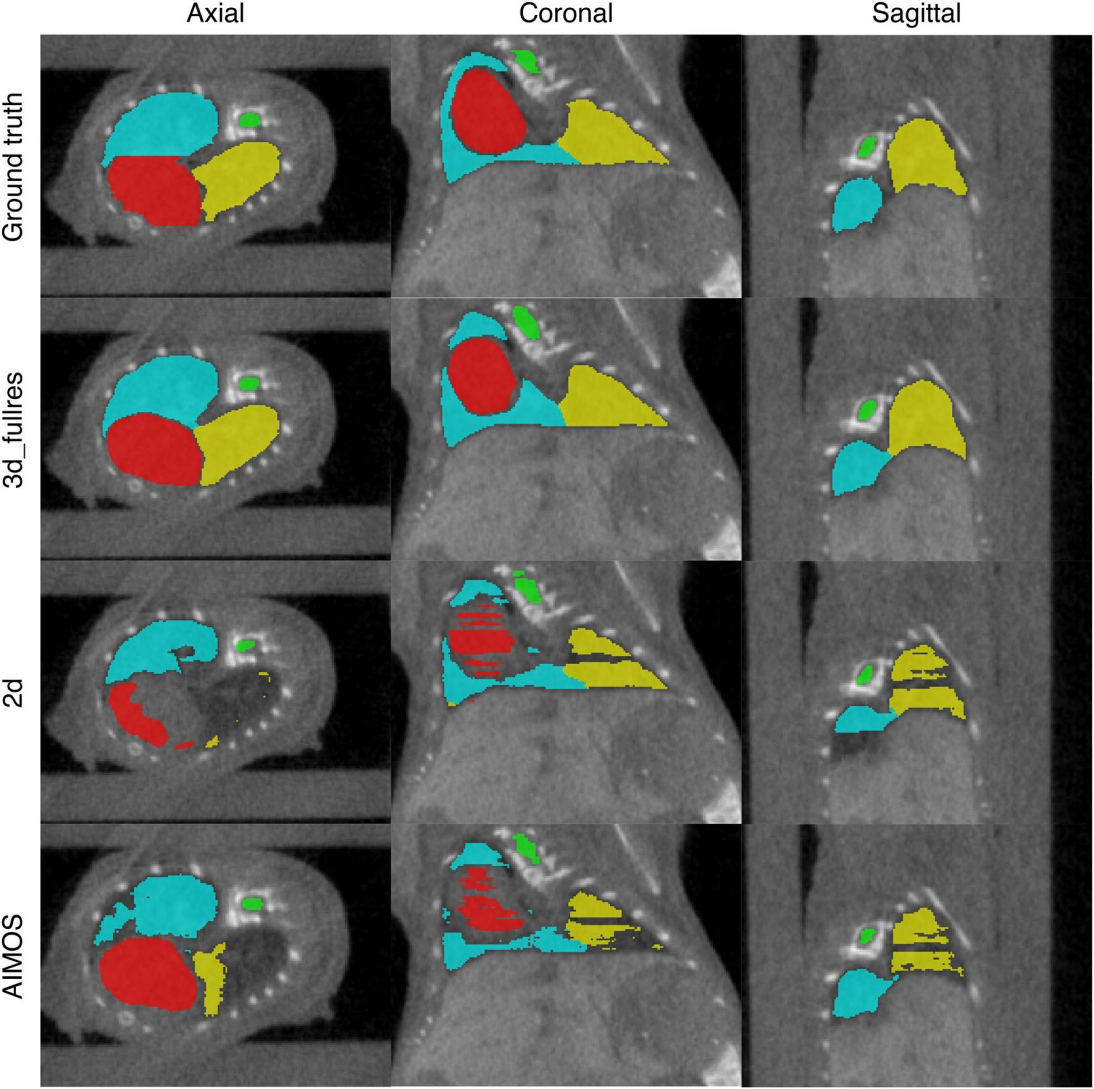
An example segmentation in the axial, coronal and sagittal views for test set 2. The first row shows the manual contours of observer 1 while the succeeding rows are the automated contours generated by each model. Contours in red, green, blue and yellow correspond to the heart, spinal cord, right lung and left lung, respectively.

Since the overall best performing models for the native CT dataset are the 3d_fullres and 3d_cascade models and no significant difference was observed between them, the 3d_fullres model was chosen as the representative 3D model to evaluate the contrast-enhanced CT dataset. The results were compared to both nnU-Net 2d and AIMOS. Figure 2 shows a visual comparison of the manual contours of observer 1 and the automated contours generated by networks trained on the native CT data. The segmentation performance of the models versus observer 1 in terms of the DSC, MSD and 95p HD are summarized in Table 2, and the corresponding boxplots are shown in Supplementary Fig. S3. For all structures, the nnU-Net 3d_fullres model produced accurate segmentations albeit with a drop in performance as compared to the native CTs. Except for the spinal cord, the 3d_fullres model achieved a mean DSC $>0.90$. The mean MSDs were also smaller than the in-plane voxel size of 0.14 mm except for the heart while all organs had a mean 95p HD below 0.60 mm.

**Figure 3:**
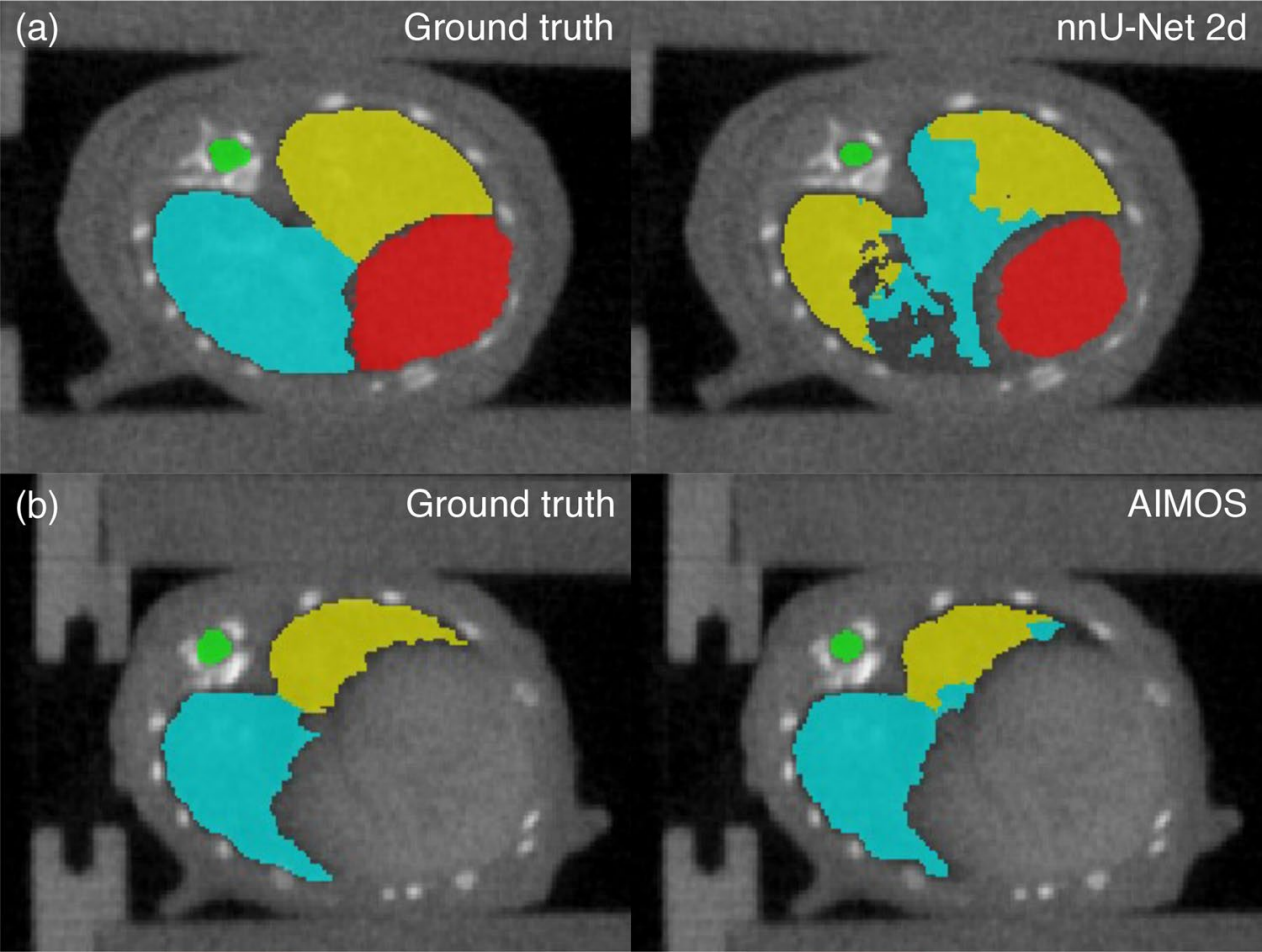
Predictions of (**a**) nnU-Net 2d and (**b**) AIMOS on contrast-enhanced CTs showing misclassification of the right and left lungs. Corresponding ground truths are given on the left. Contours in red, green, blue and yellow correspond to the heart, spinal cord, right lung and left lung, respectively.

Consistently, the AIMOS and nnU-Net 2d models exhibited greater variations in the DSC, MSD and 95p HD compared to the 3d_fullres model. Both 2D models failed to generate predictions for the heart and left lung in 1 out of 35 samples. This particular case was excluded in the calculation of the performance metrics presented in Table 2. On most of the samples, the 2D models had difficulties in segmenting the heart, right lung, and left lung. Several slices were partially or completely unlabelled for these organs and in some cases, half of the volume had no prediction at all. The lower DSC and larger MSD and 95p HD clearly indicate that the 2D models underperformed on this dataset. Notably, the 2D models scored a mean 95p HD $>1\ \hbox {mm}$ for all target organs except for the spinal cord segmentation of AIMOS. This can be attributed to mislabelling pixels in the liver as heart while pixels associated to air cavities outside the thoracic region such as air pockets in the abdomen were mislabelled as part of the lungs. Segments of the spinal cord were also missing in some cases, but it happened less frequently. Both 2D models also failed to distinguish the left and right lungs as shown in Fig. 3, which did not occur for the 3D model.

**Table 2:** Comparison of the the segmentation accuracy between different models in terms of the DSC, MSD and 95p HD (mean ± SD and median) for test set 2 annotated by observer 1.

Organ	Algorithm	Model	DSC		MSD (mm)		95p HD (mm)	
			Mean ± SD	Median	Mean ± SD	Median	Mean ± SD	Median
Heart	nnU-Net	3d_fullres	0.92 ± 0.02	0.92	0.16 ± 0.04	0.16	0.55 ± 0.18	0.54
		2d	0.81 ± 0.20	0.89*	0.36 ± 0.28	0.28*	1.48 ± 0.93	1.41*
	AIMOS	UNet-768	0.83 ± 0.17	0.89*	0.34 ± 0.24	0.27*	1.50 ± 0.86	1.29*
Spinal Cord	nnU-Net	3d_fullres	0.85 ± 0.03	0.85	0.07 ± 0.04	0.07	0.39 ± 0.11	0.40
		2d	0.76 ± 0.14	0.81*	0.19 ± 0.42	0.10*	1.29 ± 2.24	0.59*
	AIMOS	UNet-768	0.82 ± 0.05	0.83*	0.08 ± 0.02	0.08*	0.45 ± 0.13	0.40*
Right Lung	nnU-Net	3d_fullres	0.96 ± 0.01	0.96	0.06 ± 0.02	0.06	0.45 ± 0.06	0.42
		2d	0.82 ± 0.14	0.86*	0.55 ± 0.39	0.45*	6.14 ± 5.31	3.63*
	AIMOS	UNet-768	0.87 ± 0.16	0.92*	0.21 ± 0.23	0.15*	1.11 ± 1.24	0.60*
Left Lung	nnU-Net	3d_fullres	0.95 ± 0.02	0.95	0.06 ± 0.03	0.06	0.59 ± 0.17	0.56
		2d	0.77 ± 0.18	0.81*	0.80 ± 0.64	0.65*	9.54 ± 9.88	3.52*
	AIMOS	UNet-768	0.77 ± 0.22	0.87*	0.42 ± 0.89	0.17*	2.80 ± 5.29	1.12*

The asterisk (*) indicates a significant difference with the nnU-Net 3d full resolution model according to the Wilcoxon signed rank test with a significance level of $\alpha =0.05$.

### Best, intermediate, and worst segmentations

**Figure 4:**
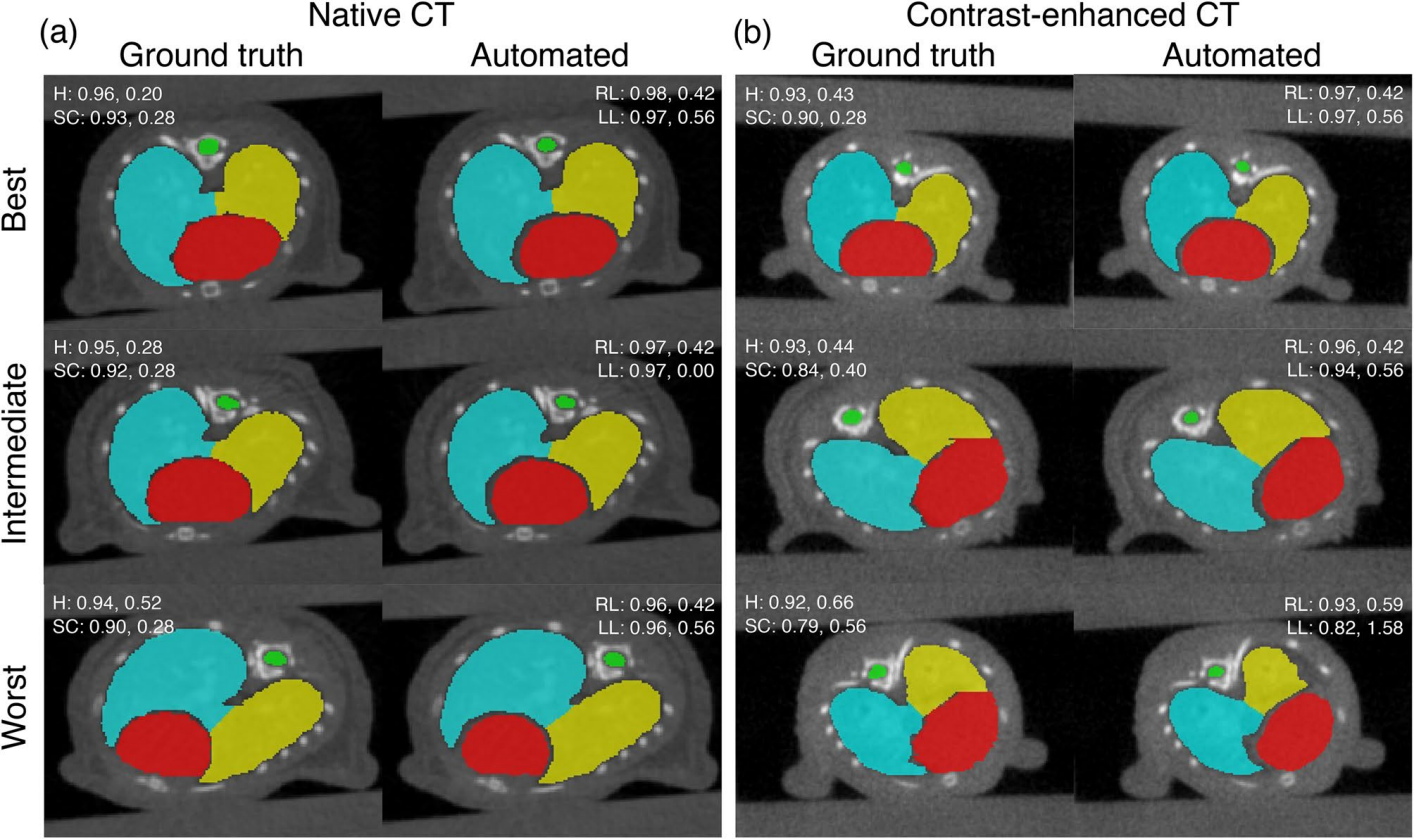
Examples of the best (first row), intermediate (second row), and worst (third row) segmentation results for the (**a**) native CT and (**b**) contrast-enhanced CT datasets obtained by the nnU-Net 3d_fullres model compared to the ground truth. The DSC scores (first value) and 95p HD in mm (second value) for each organ are also given. H, SC, RL and LL correspond to the heart (red), spinal cord (green), right lung (blue) and left lung (yellow), respectively.

The best, intermediate and worst segmentation results of the nnU-Net 3d_fullres model for both datasets are shown in Fig. 4. The samples were chosen based on the average DSC of the organs. For the native CT dataset, all three contours showed good agreement with the ground truth. For the contrast-enhanced CT dataset, the quality of the best and intermediate results are similar to the native CT. However, the left lung is undersegmented for the worst case. The model had difficulty annotating the lungs because the contrast with soft tissue is not as good as the native CTs. This particular case is actually an extreme outlier and majority of the automated contours for the contrast-enhaced CT dataset did not exhibit such errors. Unlike the native CT dataset, the worst case for the contrast-enhanced CTs required minor manual corrections. Nevertheless, the contours generated by the nnU-Net 3d_fullres model for this case are much better than AIMOS. In fact, AIMOS achieved DSC values of 0.15 (heart), 0.57 (spinal cord), 0.08 (right lung), and 0.001 (left lung), whereas nnU-Net 3d_fullres obtained DSC values of 0.88 (heart), 0.79 (spinal cord), 0.93 (right lung), and 0.82 (left lung).

It can be seen that the proposed model handles organ edges better than humans, particularly for the heart and lungs. The ragged edge details in the ground truth are partly due to the fact that the contours were created in the coronal plane. That choice was made as the organs are more visible and easier to distinguish in that plane. Unfortunately, the software does not allow editing of the contours on planes other than the one initially used to create them.

### Interobserver variability (IOV)

Table 3 gives the mean $\hbox {CI}_{\mathrm{gen}}$ of the heart, total lungs and spinal cord computed between the automated and reference contours for both test sets and Supplementary Fig. S4 shows the corresponding boxplots. The performance of nnU-Net 3d_fullres, 2d and AIMOS was evaluated against the reference established based on the interobserver variability. For the native CT dataset, all models showed comparable results and obtained higher conformity indices than the human observers for all target organs. For the contrast-enhanced CT dataset, only the 3d_fullres model achieved greater conformity than the IOV on all organs, whereas AIMOS indicated better conformity than the human baseline only for the spinal cord delineation. For both datasets, the $\hbox {CI}_{\mathrm{gen}}$ is notably lower for the spinal cord which is due to manual delineation variation in the superior and inferior extent. The small cross-section of a mouse spinal cord also made this structure difficult to delineate, resulting in disagreements along the organ boundary.

**Table 3:** Performance of the models against the reference contour in terms of the generalized conformity index ($\hbox {CI}_{\mathrm{gen}}$) (mean ± SD) for the native and contrast-enhanced CT datasets.

Algorithm	Models	Native CT (test set 1)			Contrast-enhanced CT (test set 2)		
		Heart	Total lungs	Spinal Cord	Heart	Total lungs	Spinal Cord
nnU-Net	3d_fullres	**90**% ± **1**%	**95**% ± **1**%	65% ± 6%	**87**% ± **3**%	**92**% ± **2**%	**75**% ± **7**%
	2d	89% ± 2%	94% ± 1%	**66**% ± **6**%	72% ± 22%	81% ± 16%	65% ± 14%
AIMOS	UNet-768	89% ± 2%	93% ± 1%	64% ± 6%	74% ± 18%	76% ± 18%	73% ± 8%
Interobserver variability		79% ± 3%	86% ± 2%	60% ± 6%	80% ± 3%	81% ± 8%	69% ± 6%

The model with the best results are shown in bold.

### Contouring time

Table 4 shows the preprocessing and inference time per scan and the total runtime for each model. The variation in the values presented is in the order of a few seconds. As expected, the 2D models had the best inference speeds and total runtimes. Although AIMOS is faster than nnU-Net 2d at inference, both models take similar amount of time to generate the contours. Among the 3D models, the 3d_lowres model had the shortest inference time, and it is faster by a factor of two than the 3d_fullres model. Due to the additional step of downsampling the images in preprocessing, the 3d_lowres model’s runtime is longer than the 3d_fullres model. The 3d_cascade model is the slowest as it executes the 3d_lowres model first and uses its prediction as the input for inference at full resolution in the second stage. For comparison, a trained biologist from our institute takes roughly 40 min to create the manual contours per animal whereas all models took less than 1 min.

**Table 4:** Comparison of the average preprocessing and inference times, and total runtimes in seconds.

Algorithm	Model	Preprocessing	Inference	Runtime
nnU-Net	3d_fullres	5	27	40
	3d_lowres	14	13	50
	3d_cascade (2nd stage only)	17	28	52
	2d	5	7	21
AIMOS	UNet-768	6	2	20

## Discussion

Typically, image-guided preclinical irradiations require that animals are imaged shortly before irradiation. This entails that the irradiation workflow must be executed in the shortest time possible as animals are continuously exposed to anaesthesia throughout the entire process. One aspect in which time can be effectively reduced is organ contouring. To date, among state-of-the-art methods for autocontouring of mouse organs, deep learning-based algorithms show superior results and outperform atlas-based segmentation techniques^25,26^. In this study, we further explore deep learning models, in particular 3D U-Net-like neural networks, and compare their performance to the 2D U-Net-based AIMOS, which is the current best performing algorithm for mouse organ segmentation. We trained and validated all the networks for heart, spinal cord, right lung and left lung segmentation in mice micro-CT images. We used the same micro-CT data as the AIMOS paper. However, we did not train and evaluate the networks separately on both the native and contrast-enhanced CT images. Instead, the training was performed only on the native CT images while the performance was evaluated on data drawn from the same distribution (i.e., native CT images not used in training and validation) and on out-of-distribution data (i.e., contrast-enhanced CT images). The use of a different strain and age of mouse, imaging with different exposure conditions, and addition of contrast material as in the contrast-enhanced CT dataset represent a large distribution shift from the training data. Evaluation on such dataset gives a worst case estimate of the performance when the models are deployed in routine practice. For most micro-CTs typically taken at preclinical irradiation facilities, the performance is expected to be closer to that of the native CT dataset.

The DSC, MSD and 95p HD were chosen to evaluate the segmentation accuracy of the trained networks. As expected, all neural networks provided accurate segmentations of the target organs when evaluated on micro-CTs drawn from the same distribution as the training data. The DSC and MSD scores of the 2D and 3D models were comparable, and the 95p HDs for all models were well below 1 mm, with two extreme outliers for right lung segmentation of nnU-Net 2d. However, this problem was easily corrected by applying connected component analysis since the pixels are sufficiently far from the region of interest. For preclinical irradiations, a contouring accuracy of about 1 mm is reasonable considering organ movement in the thorax. Moreover, most irradiators use a discrete set of collimators, with differences in size of 1 mm or more^2,33^. This contouring margin is also large enough to account for the penumbra (20-80%), which was reported to be around 0.5 mm for x-rays^3^ and 0.8 mm for proton beams^7^ under standard setup conditions.

Overall, the nnU-Net 3d_fullres and 3d_cascade models showed superior segmentation performance for native CTs. Since no significant difference is observed between them, and since the 3d_fullres model is faster in terms of training and inference, it was deemed the best performing model for this segmentation task. Consistent for all organs, the 3d_fullres model gives a small but significant accuracy benefit compared to AIMOS. For the contrast-enhanced CTs, however, the benefit of the proposed 3D model is very large compared to AIMOS. For instance, AIMOS exhibited unacceptably large Hausdorff distances, resulting mainly from erroneous classifications on other regions of the scan. Such errors, similar to the outliers observed for nnU-Net 2d on native CT scans, can be attributed to the loss of craniocaudal information in 2D networks. Since 2D networks are trained on individual coronal slices, Z position information is not preserved in the training, which makes them more prone to mislabelling closely resembling pixels far from the region of interest. In effect, 2D networks like AIMOS require more labor-intensive corrections, which render them less useful in practice. Even for the most difficult case in the contrast-enhanced CT dataset, the 3d_fullres model demonstrated a more stable performance than AIMOS. Therefore, for data on which the model has not been trained on, which is common when rolling this out in the field, the proposed model is much more robust and better generalizable. This is an advantage for preclinical facilities where various animal studies are conducted, which typically have different experimental designs. In such facilities, it is difficult to build a training dataset that spans all types of images that the model would face, due to restrictions on the use of animals for imaging experiments.

When evaluated on the contrast-enhanced CT images, the nnU-Net 3d_fullres model trained on the native CTs perform equally well with AIMOS trained on the contrast-enhanced CTs. As reported in the literature, AIMOS achieved a median DSC of 0.92 (heart) and 0.95 (total lungs), and median 95p HD of 0.50 mm (heart) and 0.20 mm (total lungs)^26^. These values are comparable to the median DSC of 0.92 (heart) and 0.96 (total lungs), and median 95p HD of 0.54 mm (heart) and 0.28 mm (total lungs) achieved by our model. However, when AIMOS is not retrained on the contrast-enhanced scans, its performance on those data is considerably worse. This further confirms the superiority of the nnU-Net 3d_fullres model. Aside from employing a 3D neural network, another advantage of nnU-Net is it automatically determines the training configuration such as network depth, batch size, patch size, learning rate, and class sampling strategy tailored to the dataset provided by the user and thus removing the burden of manual tuning. It also employs more extensive data augmentation techniques than AIMOS.

To further establish the usefulness of the models for autocontouring in routine practice, they must maintain good agreement with expert contours. For that, we compared the conformity indices of the nnU-Net 3d_fullres, 2d and AIMOS models to a consensus segmentation and evaluated their performance against the interobserver variability (IOV) for the heart, total lungs and spinal cord. Although all three models showed superior results to the IOV for the native CT dataset, only the 3d_fullres model showed higher conformity indices on all target organs for the contrast-enhanced CT dataset. These results indicate the possibility that the 3d_fullres model is better than humans. However, further research is needed to establish this claim. A possible follow up can be a blind scoring study where participants are asked to select their preferred segmentation between manual and automated contours.

Lastly, to determine the impact of integrating these autocontouring tools in the preclinical workflow, we also measured the inference time and total runtimes of the models. Using our computing system, AIMOS achieved the fastest inference time at 2 s whereas nnU-Net 3d_fullres model took 27 s to generate predictions. The average runtime per animal for the proposed 3D model is 40 s. This runtime includes preprocessing and loading of the micro-CT images, making inference, and exporting the final contours to the desired format. Although we expect this runtime can be substantially shortened with better implementation, it is already a significant improvement from the manual contouring time of about 40 min per animal for this particular segmentation task.

This work has demonstrated that nnU-Net deep learning pipeline can be used and integrated into the preclinical workflow to provide fast and accurate contouring. While more advanced network architectures may be of interest of study, our results with nnU-Net, which employs a generic U-Net architecture, showed that the whole training process is equally important to achieve good performance across datasets. In the future, we intend to assess this method to other treatment sites and imaging modalities. In preclinical studies, MRI scans of the brain and head-and-neck are of particular interest since micro-CTs have poor contrast in these regions.

## Conclusions

In summary, we reported the segmentation performance, generalizability and efficiency of nnU-Net and AIMOS for autocontouring of the heart, spinal cord, right lung and left lung in mice micro-CT images. The best performing model for this segmentation task is the nnU-Net 3d_fullres model which is capable of generating high quality segmentations across diverse datasets while maintaining good levels of agreement with expert contours. It also offers significant improvement in countouring time. Its implementation in routine practice as an autocontouring tool can potentially expedite the preclinical workflow and reduce the overall workload.

## Supplementary Information

Supplementary Information.

## Data Availability

Annotations of the heart, spinal cord, right and left lungs used for training and testing are publicly available at https://doi.org/10.5281/zenodo.5121272. The pre-trained nnU-Net 3d_fullres model is accessible at https://doi.org/10.5281/zenodo.5786839.
