# Supplementary material for: Deep learning-based segmentation of the thorax in mouse micro-CT scans
Source: Sci Rep. 2022 Feb 2;12:1822. doi: 10.1038/s41598-022-05868-7 (PMC8810936; doi:10.1038/s41598-022-05868-7)
Supplement: Supplementary file 1 — Supplementary Information. [file 41598_2022_5868_MOESM1_ESM.pdf]

Deep learning-based segmentation of the thorax in mouse micro-CT scans

Justin Malimban[1,*], Danny Lathouwers[2], Haibin Qian[3], Frank Verhaegen[4], Julia Wiedemann[1,5], Sytze Brandenburg[1,+], and Marius Staring[6,+]

[1]Department of Radiation Oncology, University of Groningen, University Medical Center Groningen, Groningen, 9700 RB, The Netherlands
[2]Department of Radiation Science and Technology, Faculty of Applied Sciences, Delft University of Technology, Delft, 2629 JB, The Netherlands
[3]Department of Medical Biology, Amsterdam University Medical Centers (location AMC) and Cancer Center Amsterdam, 1105 AZ, The Netherlands
[4]Department of Radiation Oncology (MAASTRO), GROW School for Oncology and Developmental Biology, Maastricht University Medical Center, Maastricht, 6229 ER, The Netherlands
[5]Department of Biomedical Sciences of Cells & Systems-Section Molecular Cell Biology, University of Groningen, University Medical Center Groningen, Groningen, 9700 RB, The Netherlands
[6]Department of Radiology, Leiden University Medical Center, Leiden, 2333 ZA, The Netherlands
[*]j.malimban@umcg.nl
[+]these authors contributed equally to this work

Supplementary figures

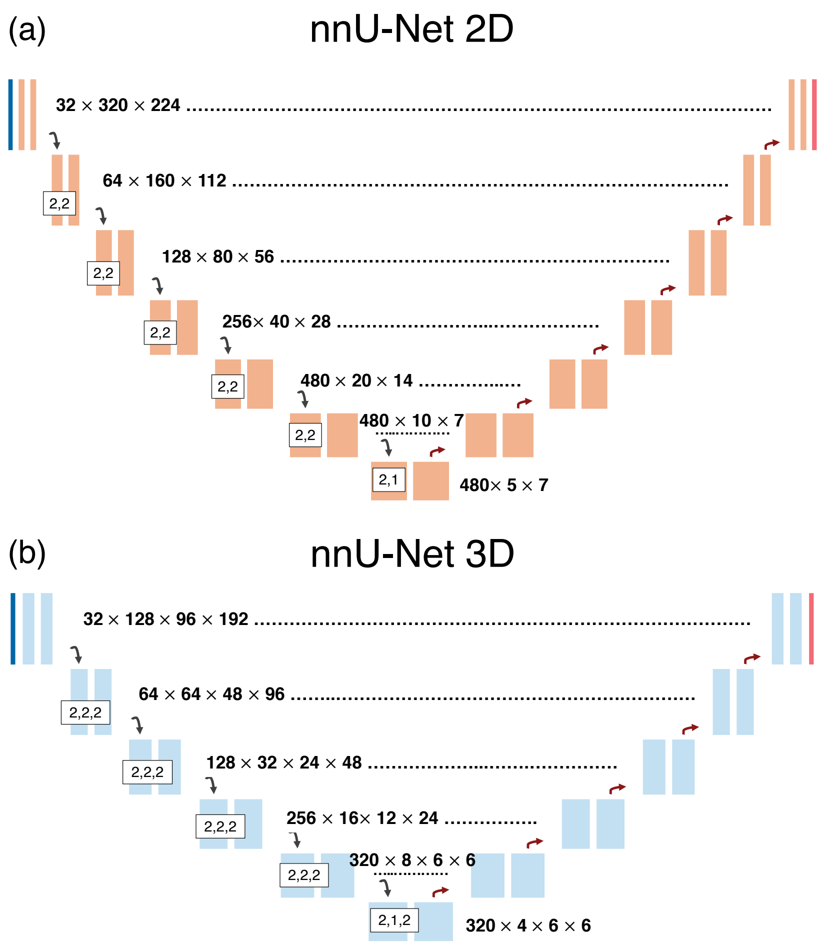

**Dataset**	
[0.5, 99.5] percentile	[-664, 277]
Mean	-224.7
Standard deviation	258.9
**2D U-Net**	
Target spacing	0.14 × 0.14
Median Shape @ Target Spacing	284 × 210
Patch Size	320 × 224
Batch Size	44
**3D full resolution U-Net**	
Target spacing	0.14 × 0.14 × 0.14
Median Shape @ Target Spacing	284 × 210 × 405
Patch Size	128 × 96 × 192
Batch Size	2
**3D low resolution U-Net**	
Target spacing	0.19 × 0.19 × 0.19
Median Shape @ Target Spacing	207 × 153 × 295
Patch Size	128 × 96 × 192
Batch Size	2

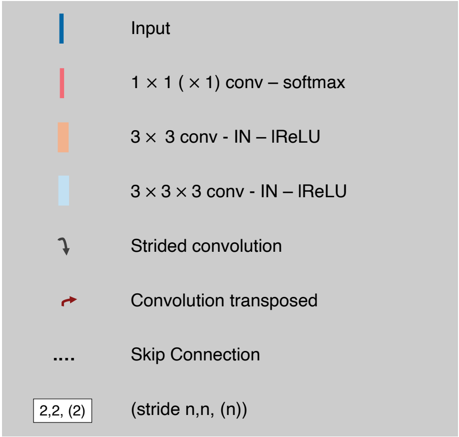

Figure S1. (a) 2D and (b) 3D U-Net architectures generated by nnU-Net for this dataset. The orange and blue boxes correspond to a convolution (conv)-instance normalization (IN)-leaky ReLU (lReLU) unit in 2D and 3D, respectively. The numbers beside each encoder level correspond to the channel × image size in x, y, (z) at that stage. Gray arrows indicate strided convolutions while red arrows denote convolution transposed. The final layer uses a 1 × 1 (× 1) convolution and a softmax activation function. The table in the upper right gives the properties of the foreground classes used for preprocessing the images (under Dataset). The target spacing, median shape at target spacing, patch size and batch size for the nnU-Net 2d, 3d_fullres and 3d_lowres models are also given.

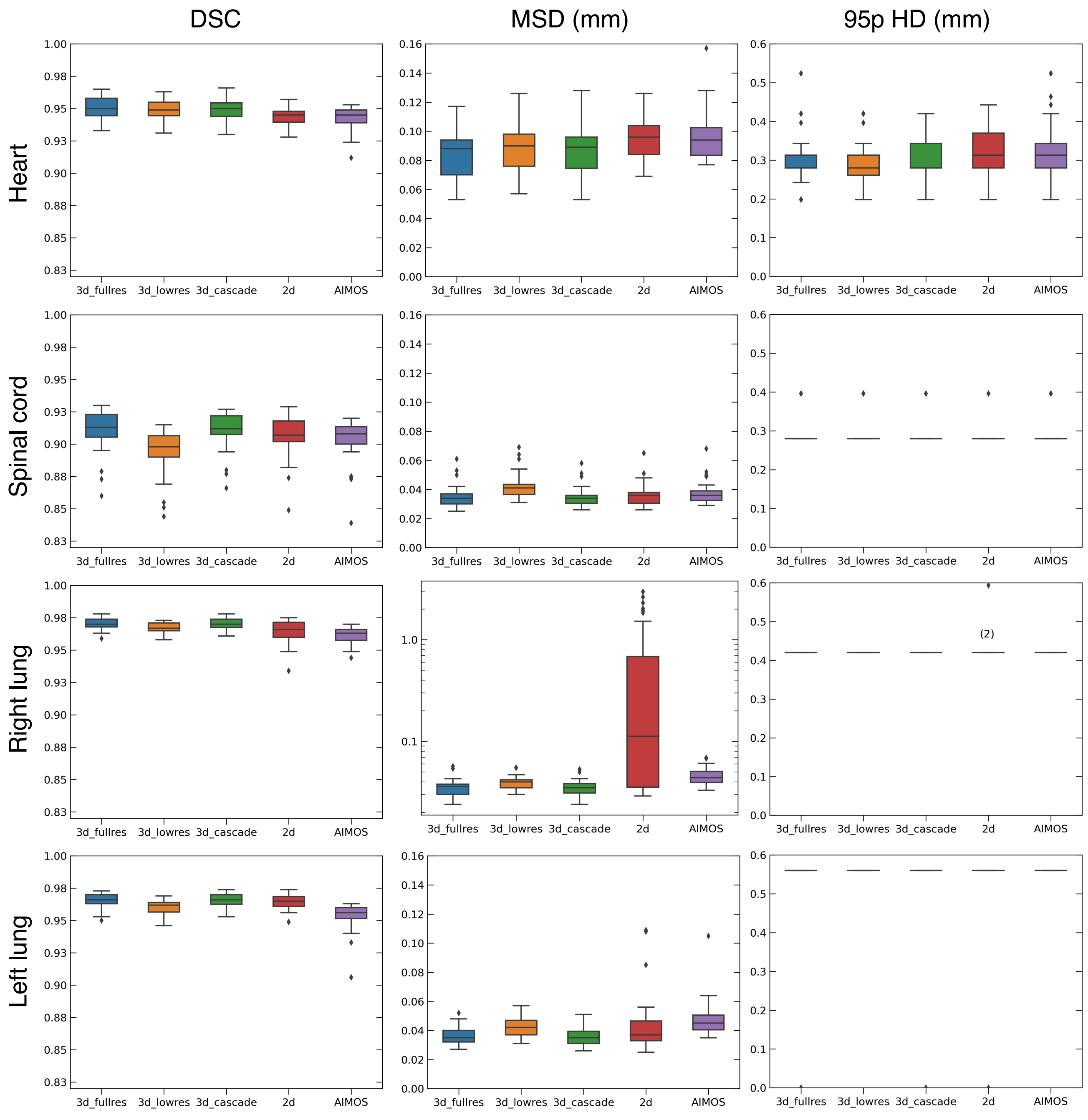

Figure S2. Boxplots of the evaluation metrics for test set 1 (native CTs). The first, second and last columns correspond to the DSC, MSD and 95p HD, respectively. The number above the 95p HD boxplot of the nnU-Net 2d model for the right lung indicates the number of images with a value higher than the maximum y-value in the plot. Tight 95p boxplots for the spinal cord, right and left lungs were observed due to very little variation over the dataset. Legend: box = interquartile range, line = median, whiskers = minimum and maximum and diamond = outliers.

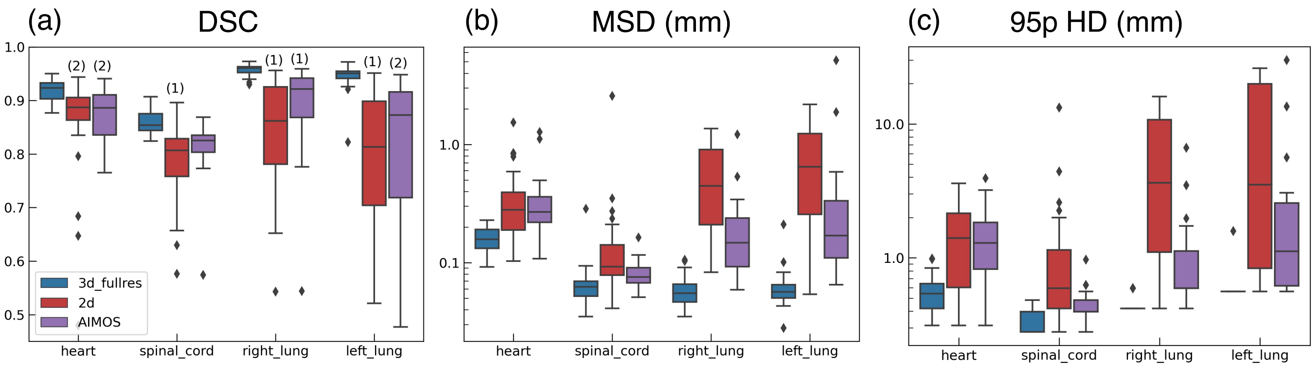

Figure S3. Boxplots of the evaluation metrics for test set 2 (contrast-enhanced CTs). The plots on the left, middle and right correspond to the DSC, MSD and 95p HD, respectively. The numbers above the DSC boxplots indicate the number of images with a value lower than the minimum y-value. Legend: box = interquartile range, line = median, whiskers = minimum and maximum and diamond = outliers.

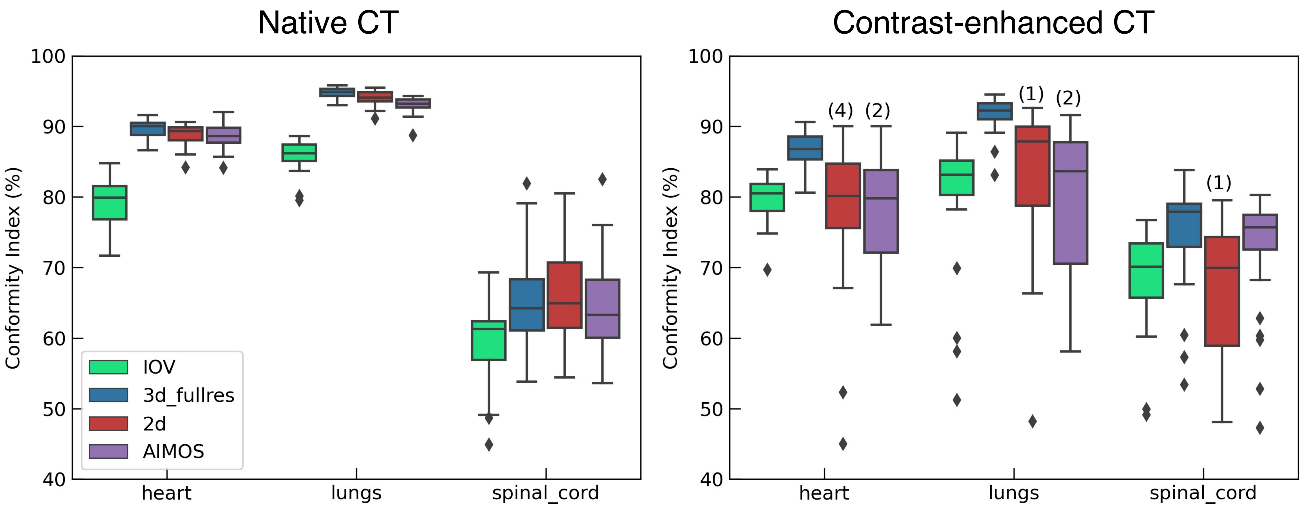

Figure S4. Boxplots of the conformity index between the models and the reference contours for (a) test set 1 and (b) test set 2. The conformity index among human observer delineations (green) was the baseline for comparison. The numbers above the boxplots indicate the number of images with a value lower than the minimum y-value. Legend: box = interquartile range, line = median, whiskers = minimum and maximum and diamond = outliers.

Supplementary tables

Dataset	Scanner	#Animals/ images	Category	Min and max image size [x,y,z]	Voxel spacing [mm^3]
Native CT	Tomoscope Duo	15/105	train/ validation	280×202×371, 290×224×421	0.14×0.14×0.14
		5/35	test set 1	281×207×531, 290×220×603	
Contrast-enhanced CT	InSyte	10/35	test set 2	308×242×484	0.14×0.14×0.14

Table S1. Properties of the mouse datasets used in this study.
